# Root Transcriptome Analysis Identifies Salt-Tolerance Genes in Sweet Corn Chromosome Segment Substitution Lines (CSSLs)

**DOI:** 10.3390/plants14111687

**Published:** 2025-05-31

**Authors:** Zili Zhang, Xuxuan Duan, Pengfei Liu, Qingchun Chen, Wei Sun, Xiaorong Wan, Yixiong Zheng, Jianting Lin, Feng Jiang, Faqiang Feng

**Affiliations:** 1 Guangzhou Key Laboratory for Research and Development of Crop Germplasm Resources, College of Agriculture and Biology, Zhongkai University of Agriculture and Engineering, Guangzhou 510225, China; zilizhang0501@zhku.edu.cn (Z.Z.); 17868869627@163.com (X.D.); lpf2004buildit@aliyun.com (P.L.); chenqingchun0414@163.com (Q.C.); sunwei0211@zhku.edu.cn (W.S.); biowxr@126.com (X.W.); gdsscqs@163.com (Y.Z.); 2 Guangdong Provincial Key Laboratory of Plant Molecular Breeding, College of Agriculture, South China Agricultural University, Guangzhou 510642, China; 13539328142@163.com

**Keywords:** sweet corn, salt stress, transcriptome, reactive oxygen species, homeostasis, hormone signaling

## Abstract

Salt stress severely constrains global crop productivity. However, most sweet corn cultivars exhibit weak tolerance to salt stress. In this study, two sweet corn CSSLs, salt-tolerant line D55 and salt-sensitive line D96, were selected as materials. We conducted comparative phenotyping and physiological profiling of seedlings under salinity treatment, and transcriptome analysis was carried out by sampling root tissues at 0 h, 4 h, 12 h, and 72 h post-treatment. The results indicated that D55 exhibited enhanced seedling height, root length, fresh weight, relative chlorophyll content, and antioxidant enzyme activities, while showing reduced malondialdehyde accumulation in comparison to D96. Pairwise comparisons across time points (0 h, 4 h, 12 h, 72 h) identified 6317 and 6828 differentially expressed genes (DEGs) in D55 and D96. A total of 49 shared DEGs across four time points were identified in D55 and D96, which were enriched in 12 significant Gene Ontology (GO) terms. Only eight DEGs were shared between genotypes across all comparisons. Transcriptomic analysis revealed 1281, 1946, and 1717 DEGs in genotypes D55 and D96 at 4 h, 12 h, and 72 h post-salt treatment, respectively. Genes associated with reactive oxygen species (ROS) homeostasis, phenylpropanoid metabolism, cutin, suberin and wax biosynthesis, and benzoxazinoid synthesis exhibit enhanced sensitivity in the salt-tolerant genotype D55. This leads to an enhanced ROS scavenging capacity and the establishment of a multi-layered defense mechanism. Additionally, brassinosteroid (BR), gibberellin (GA), and abscisic acid (ABA) and auxin-related genes exhibited different responses to salt stress in sweet corn. A hypothetical model, which established a multi-layered salt adaptation strategy, by integrating ROS detoxification, osmotic balance, and phytohormone signaling, was put forward. By integrating transcriptome and differential chromosomal fragment data, our findings identify 14 candidate genes for salt tolerance, providing potential ideal target genes in breeding to improve salt tolerance in sweet corn.

## 1. Introduction

Salinity stress poses a major constraint to global agricultural sustainability and food security [1,2]. Currently, over 800 million hectares of land worldwide are affected by soil salinization, representing approximately 6% of the Earth’s total land area and 20% of cultivated lands [3,4]. This issue is exacerbated by insufficient rainfall, improper irrigation practices using saline water, and accelerating climate change, with projections indicating significant expansion of salt-affected areas in the coming decades [5]. Arid and semi-arid regions with high evapotranspiration rates are particularly vulnerable to secondary salinization [6], while coastal zones face increasing soil salinity due to sea level rise and saltwater intrusion [7]. This trend has a profound and sustained impact on the production systems of staple crops, including rice, wheat, and maize. Salt stress has emerged as a critical bottleneck constraining global food security.

Salinity stress represents a major abiotic constraint limiting plant growth and agricultural productivity, disrupting cellular homeostasis through multiple mechanisms including osmotic imbalance, ion toxicity, and oxidative damage [4]. Root systems serve as the primary organ for direct salt stress response in plants. Excessive sodium ion (Na^+^) accumulation under saline conditions perturbs Na^+^/K^+^ balance and impairs cellular metabolism [8]. To counteract this toxicity, plants employ dual strategies: Na^+^ exclusion through root barriers and vacuolar sequestration via membrane-bound transporters [9]. The tonoplast Na^+^/H^+^ antiporters play critical roles in compartmentalizing Na^+^ into vacuoles, preventing cytotoxic accumulation [10]. Notably, the SOS (Salt Overly Sensitive) signaling pathway coordinates ion homeostasis through plasma membrane Na^+^ extrusion systems [11]. Oxidative stress constitutes another critical challenge, as salinity induces excessive reactive oxygen species (ROS) production, leading to cellular damage and programmed cell death [12]. Plants activate comprehensive antioxidant defenses encompassing enzymatic systems (e.g., superoxide dismutase, catalase) and non-enzymatic scavengers (e.g., glutathione, flavonoids) to mitigate ROS toxicity [13,14]. Comparative studies reveal that salt-tolerant species typically exhibit enhanced antioxidant capacity and efficient ROS detoxification mechanisms [15]. In summary, plants address the challenge of salt stress through a diverse range of regulatory mechanisms.

Multiple phytohormones play pivotal roles in salinity tolerance mechanisms [16]. Auxin orchestrates root architecture modifications, which stimulates cell division and root remodeling to improve stress resilience [17]. Abscisic acid (ABA) serves as a key signaling molecule, inducing stomatal closure to reduce transpirational water loss while upregulating antioxidant enzyme activity to mitigate oxidative damage [18]. Cytokinins maintain meristematic activity under saline conditions through cell cycle regulation and growth promotion [19]. Jasmonate biosynthesis genes show tissue-specific upregulation during salt stress, particularly in the leaves and roots, leading to elevated endogenous jasmonic acid levels [20]. Gibberellins coordinate developmental plasticity and osmotic adjustment, facilitating environmental adaptation through growth homeostasis regulation [21,22]. These hormonal networks synergistically modulate physiological processes including ion transport, osmotic balance, and stress signaling. Although significant advancements have been achieved in the study of salt stress, the underlying molecular mechanism remains to be elucidated.

Multi-omics technologies have advanced mechanistic insights into maize salt stress responses, identifying DEGs and pathways (MAPK signaling, phytohormone transduction, carbohydrate metabolism, and flavonoid biosynthesis) via transcriptomics and RNA-seq [23,24,25,26]. Integrated analyses, including WGCNA, further pinpointed *Zm00001eb155540* as a salinity-responsive hub gene [27]. Significant progress has been made in identifying salt-tolerance genes in maize. Luo et al. [2] identified the functional roles of *SAG4* and *SAG6* in enhancing plant salt resistance. In a subsequent study, 83 single-nucleotide polymorphisms (SNPs) were associated with salinity tolerance, and two candidate genes, *ZmCLCg* and *ZmPMP3*, critically regulate ion homeostasis under salt stress [28]. Liang et al. [29] further identified 10 candidate genes linked to salt-induced osmotic stress tolerance, with METO (Membrane Electrostatic Potential Oscillator) abundance serving as a key biomarker for cellular stress adaptation. Zhang et al. [30] revealed that *ZmHKT1* encodes a high-affinity K^+^ transporter, where loss-of-function alleles result in excessive Na^+^ accumulation in the leaves and heightened salt sensitivity. In addition, eight promoter region variations correlated with root diameter under salinity were identified in the *ZmHKT1* gene, and Hap2 likely enhances salt tolerance by optimizing Na^+^ translocation efficiency [31].

This study employed a chromosome segment substitution line (CSSL) population to investigate the phonotypes under salinity stress. Two CSSLs, salt-tolerant D55 and salt-sensitive D96, were identified to minimize confounding genetic background effects. Subsequently, we conducted comparative phenotyping and physiological profiling of seedlings under salinity treatment, and transcriptome analysis was carried out by sampling root tissues at 0 h, 4 h, 12 h, and 72 h post-treatment. This study will provide novel insights into the molecular mechanism of salt stress adaptation in sweet corn.

## 2. Results

### 2.1. Differential Seedling Responses to Salt Stress Between D55 and D96

**Figure 1:**
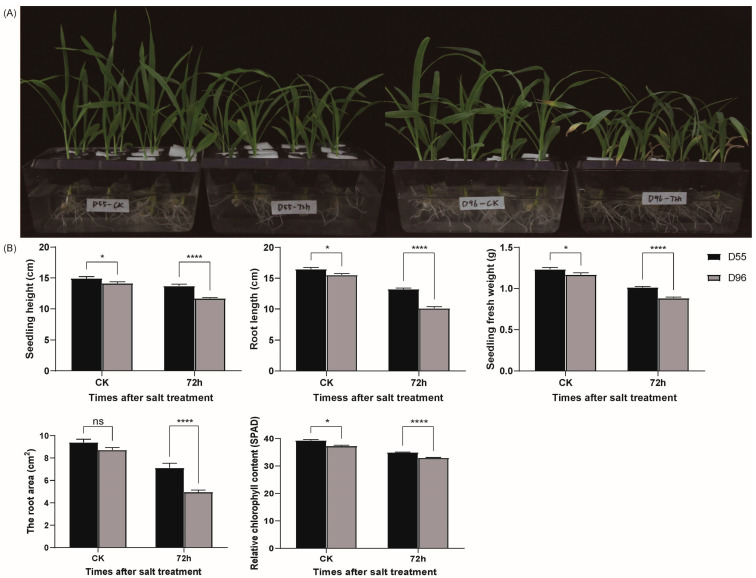
Physiological responses of sweet corn genotypes D55 and D96 to salt stress. (**A**) Phenotypic changes. (**B**) Quantitative analysis of growth parameters. Data are presented as mean ± SE (*n* = 10 biological replicates). Differences between genotypes were analyzed using Dunnett’s test (ns, not significant; * *p* < 0.05; **** *p* < 0.0001).

Distinct growth responses to salt stress were observed between the salt-tolerant line D55 and salt-sensitive line D96 at 72 h post-treatment. The D96 seedlings exhibited leaf senescence and marginal necrosis under salt stress (Figure 1A), whereas D55 maintained stable leaf morphology. In control conditions, D55 and D96 showed significant differences (*p* < 0.05) in seedling height, root length, seedling fresh weight, and relative chlorophyll content (SPAD value), but not in root surface area (Figure 1B). Under salt stress, all five parameters differed markedly between the genotypes (*p* < 0.0001), with D96 displaying greater reductions, in terms of seedling height (14.65%), root length (23.37%), fresh weight (12.92%), root surface area (30.34%), and chlorophyll content (5.59%), compared to D55 (Figure 1B). These results confirm D55’s superior salt tolerance during early seedling development.

### 2.2. Enhanced ROS Scavenging Capacity in Salt-Tolerant D55

**Figure 2:**
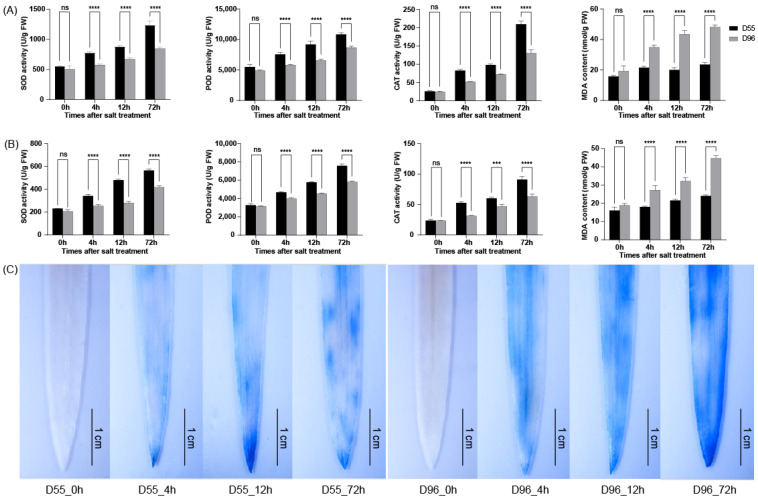
ROS scavenging dynamics in D55 and D96 under salt stress. Time-course changes in antioxidant enzyme activities and MDA levels in leaves (**A**) and roots (**B**). (**C**) Trypan blue staining showing membrane integrity. Data represent mean ± SE (*n* = 3). Dunnett’s test was used for the comparative analysis between D55 and D96. Asterisks denote significant differences between D55 and D96 (ns, not significant; *** *p* < 0.001; **** *p* < 0.0001).

The activities of antioxidant enzymes (SOD, POD, and CAT) and MDA levels were measured in the leaves and roots. Both genotypes showed time-dependent increases in SOD, POD, and CAT activities under salt stress (Figure 2A,B and Table S1). However, D55 exhibited significantly higher antioxidant enzyme activities than D96 at 4, 12, and 72 h post-treatment (Figure 2A,B). Concurrently, D55 accumulated 18–32% less MDA in leaves and roots compared to D96 during these intervals (Figure 2A,B), indicating reduced oxidative damage. While leaf MDA levels in D55 peaked at 72 h, root MDA content progressively increased throughout the stress period. In contrast, D96 displayed accelerated MDA accumulation in both tissues, correlating with its severe oxidative stress symptoms.

Histochemical staining further validated these findings. Trypan blue staining revealed progressive membrane damage in both genotypes under salt stress, but D96 leaves exhibited more intense blue coloration than D55 (Figure 2C), consistent with greater ROS-induced cell death. The delayed membrane damage in D55 aligns with its robust antioxidant enzyme system (Figure 2A,B), supporting its enhanced capacity to mitigate oxidative stress during salt exposure.

### 2.3. Transcriptomic Profiling Under Salt Stress

After salt stress treatment, transcriptome data were generated for the D55 and D96 genotypes to elucidate the adaptive strategies employed by salt-tolerant genotypes (D55). The sequencing data for each sample ranged from 5.8 to 8.3 Gb, and the clean reads were mapped to the maize genome B73 v5.0, achieving a gene region alignment rate of 95.25–96.52%. This indicates high-quality RNA sequencing (Table S2). Principal component analysis of mRNAs accurately divided all the samples into two different clusters, reflecting the obvious difference between D55 and D96 (Figure S1). To validate the DEGs identified through RNA sequencing, six DEGs at 4 h and six DEGs at 12 h were randomly selected for RT-qPCR analysis. Regression analysis demonstrated a highly significant correlation between the log_2_FC values calculated via RNA-Seq and those obtained through qRT-PCR, confirming that the qRT-PCR expression patterns align with the RNA-seq results. These findings validate the reliability and reproducibility of the RNA-seq data (Figure S2).

### 2.4. Dynamic Identification of Salt-Responsive DEGs

**Figure 3:**
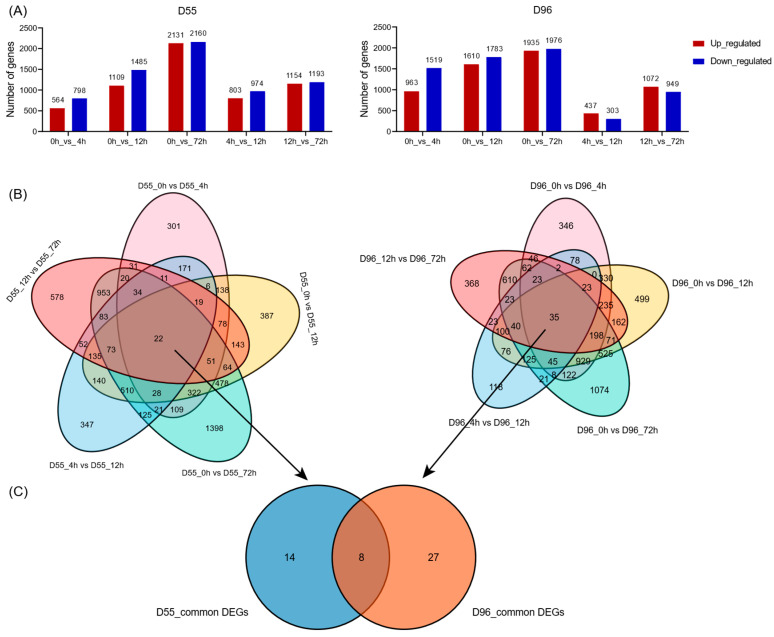
Summary of changes in transcriptome in sweet corn seedlings under salt treatment. (**A**) Summary of significant changes in the number of transcripts between different time points in D55 and D96. (**B**) Venn diagrams depicting differentially expressed genes (DEGs) in D55 and D96. (**C**) Venn diagrams depicting common differentially expressed genes (DEGs) in D55 and D96.

Pairwise comparisons across time points (0 h, 4 h, 12 h, 72 h) identified 6317 and 6828 salt-responsive DEGs (FDR < 0.05, |log_2_FC| > 1.0) in D55 and D96, respectively (Figure 3A,B; Tables S3 and S4). Temporal analysis revealed maximal DEG numbers between 0 h vs. 72 h with 4218 and 4901 DEGs in D55 and D96, respectively, and minimal changes between 4 h vs. 12 h with 512 and 687 DEGs in D55 and D96, respectively. The numbers of shared DEGs across four time points were 22 and 35 in D55 and D96, respectively. Only 8 DEGs were shared between genotypes across all comparisons, while 14 genotype-specific DEGs were exclusive to salt-tolerant D55 (Figure 3C; Table S5). Intriguingly, progressive accumulation of upregulated and downregulated DEGs occurred in both genotypes over time (Figure 3A).

### 2.5. Identification of Core DEGs and Functional Enrichment

The eight shared core DEGs across genotypes included six annotated loci: *Zm00001eb148070* (regulator of chromosome condensation 1), *Zm00001eb367810* (putative regulator of chromosome condensation (RCC1) family protein), *Zm00001eb228670* (MYB transcription factor 96), *Zm00001eb268600* (extensin-like protein), *Zm00001eb372500* (HVA22-like protein a), and one receptor-like kinase (*Zm00001eb424770*) (Table S5). Notably, two cysteine-rich receptor-like kinase genes (*Zm00001eb106380* and *Zm00001eb106390*) comprised 14.3% of D55-specific DEGs. GO enrichment analysis of 49 shared DEGs identified across all comparisons in D55 and D96 revealed 12 significant metabolic pathways (FDR < 0.05). These pathways include extracellular region, cofactor binding, coenzyme binding, and pyridoxal phosphate binding, among others (Table S6), indicating that these processes are fundamental to early salt adaptation.

### 2.6. Identification of Salt-Responsive DEGs

**Figure 4:**
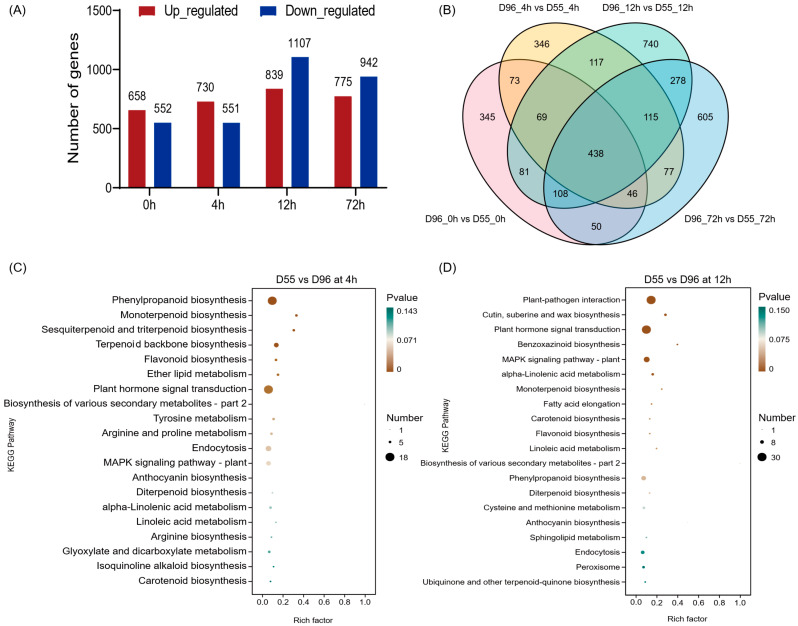
DEGs and functional enrichment between D55 and D96. (**A**) Temporal dynamics of DEGs after 0 h, 4 h, 12 h, and 72 h salt treatment. (**B**) Venn diagram illustrating genotype-specific DEG distributions. (**C**) KEGG pathway enrichment of DEGs at 4 h post-treatment. (**D**) KEGG pathway enrichment of DEGs at 12 h post-treatment.

Transcriptomic analysis revealed 1210, 1281, 1946, and 1717 DEGs in genotypes D55 and D96 at 0 h, 4 h, 12 h, and 72 h post-salt treatment, respectively (Figure 4A,B, Table S7). GO enrichment demonstrated distinct temporal regulation patterns (Figure S3). At 0 h post-salt treatment, DEGs were significantly enriched in 28 GO terms, which were predominantly associated with oxidoreductase activity (GO:0016491, GO:0016705) and iron ion binding (GO:0005506). At 4 h post-salt treatment, 55 enriched GO terms were enriched, which included oxidation–reduction processes (GO:0055114), terpenoid biosynthesis/metabolism (GO:0016114, GO:0008299), and defense responses (GO:0006952). At 12 h post-salt treatment, DEGs were enriched in 44 GO terms, which were linked to transcriptional regulation (GO:0006355, GO:0019219), macromolecule biosynthesis (GO:0010556), and redox homeostasis (GO:0055114), etc. At 72 h post-salt treatment, a total of 1717 DEGs were identified across 27 GO terms. Notably, pathways related to ROS metabolism were predominant, with significant enrichment observed in hydrogen peroxide catabolism (GO:0042744), detoxification processes (GO:0042737, GO:0097237), and responses to oxidative stress (GO:0009636). The pathway consistently enriched across all four time points was the oxidation–reduction process (GO:0055114). Additionally, the pathways that were commonly enriched at the three time points of 4, 12, and 72 h of salt treatment included the extracellular region (GO:0005576), cell wall (GO:0005618), external encapsulating structure (GO:0030312), and cell periphery (GO:0071944).

KEGG analysis revealed temporal-specific pathway activation patterns under salt stress (Figure 4C,D and Figure S3E,F). While no significantly enriched pathways were detected at 0 h, few enriched KEGG pathways emerged at later stages. At 4 h post-treatment, DEGs were enriched in phenylpropanoid biosynthesis (zma00940), monoterpenoid (zma00902), sesquiterpenoid/triterpenoid (zma00909), and terpenoid backbone biosynthesis (zma00900). At 12 h post-treatment, dominant pathways included plant–pathogen interaction (zma04626), cuticular wax biosynthesis (zma00073), hormone signaling (zma04075), and MAPK cascade regulation (zma04016). At 72 h post-treatment, sustained enrichment occurred exclusively in phenylpropanoid biosynthesis (zma00940), suggesting its persistent role in stress mitigation.

### 2.7. Enhanced ROS Scavenging and Transport Capacity in Salt-Tolerant Genotype D55

To further explore the changes in ROS related genes, we analyzed the DEGs that contribute to ROS homeostasis in the transcriptome data (Table 1). The ROS production gene *PAO* (*Zm00001eb070310*) was highly expressed in D55 at 4 h after salt stress treatment. The *RBOH* gene (*Zm00001eb341910*) was highly expressed in the sensitive line D96 at 4, 12, and 72 h after salt stress treatment, and *Zm00001eb341910* was highly expressed in D96 at 4 h after salt stress. This distinct temporal activation pattern indicates accelerated ROS production in D96 compared to D55, aligning with D96’s sensitive phenotype characterized by oxidative damage accumulation (Figure 2C). The persistent RBOH activation in D96 suggests more accumulation of ROS, exacerbating its vulnerability to salt stress.

Transcriptomic profiling revealed superior ROS management in D55 through coordinated regulation of antioxidant systems. Three *SOD* isoforms (*Zm00001eb378880*, Zm00001eb226600 and *Zm00001eb420210*) exhibited sustained upregulation in D55 across all time points. Six *GST* genes (*Zm00001eb141080*, *Zm00001eb002780*, *Zm00001eb402630*, *Zm00001eb404560*, *Zm00001eb021720* and *Zm00001eb021620*) were downregulated, showing activation in D96. *Peroxidase13* (*Zm00001eb222560*) displayed continuous upregulation in D55 at 4, 12, and 72 h after salt stress, while *Zm00001eb282430* and *Zm00001eb047120* were upregulated at 72 and 12 h, respectively. Two *POD* genes (*Zm00001eb330530* and *Zm00001eb225230*) were downregulated. Trx2 (*Zm00001eb201870*) exhibited downregulated at 72 h after salt treatment. These results are in agreement with the high activity of ROS antioxidant enzymes observed in D55 (Figure 2A,B), indicating that D55 possesses a more efficient ROS clearance system compared to D96. Additionally, *plasma membrane intrinsic protein 1* (*Zm00001eb074210*) exhibited constitutive overexpression in D55 compared to D96 throughout the stress period. These findings further substantiate that D55 exhibits superior ROS clearance and transport capabilities.

### 2.8. Upregulation of Phenylpropanoid and Benzoxazinoid Biosynthetic Pathways in D55

**Figure 5:**
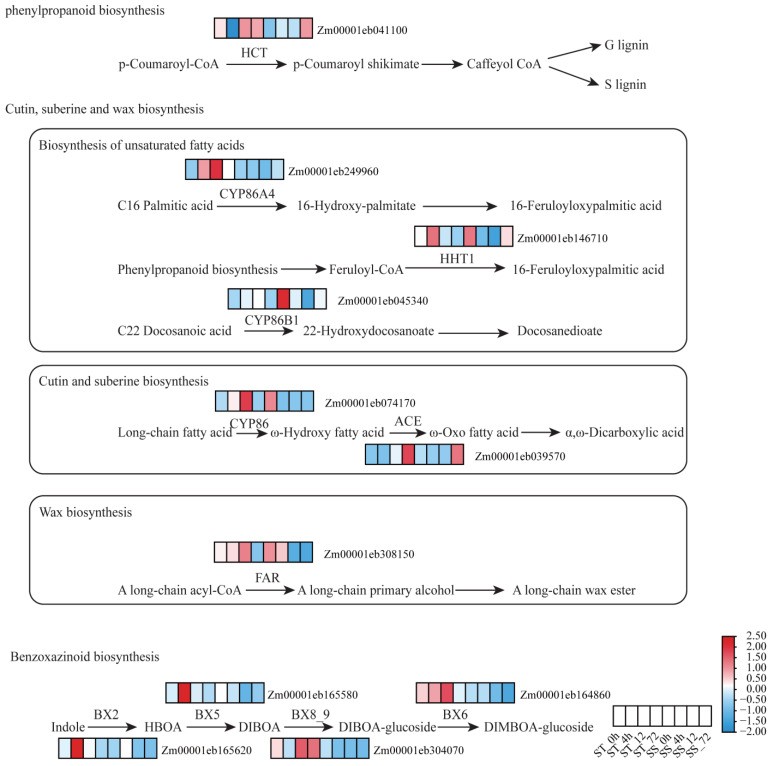
Changes in DEGs involved in phenylpropanoid biosynthesis, cutin, suberine, and wax biosynthesis, and benzoxazinoid biosynthesis in salt-tolerant D55 compared with salt-sensitive D96. A threshold of FDR < 0.05 and |log_2_FC| > 1 was used to screen DEGs between D55 and D96 after salt stress. These eight squares from left to right represent D55_0h, D55_4h, D55_12h, D55_72h, D96_0h, D96_4h, D96_12h, and D96_72h, respectively. Red and blue indicate relatively low and high expression levels (FPKM, data scaled) in comparisons between D55 and D96 after salt treatment, respectively.

DEGs between D55 and D96 were enriched in phenylpropanoid biosynthesis (zma00940), cutin, suberine, and wax biosynthesis (zma00073), and benzoxazinoid biosynthesis (zma00402) pathways (Figure 5). Key regulatory mechanisms were identified. In the phenylpropanoid biosynthesis pathway, gene *ZmHCT* (*Zm00001eb041100*) exhibited upregulation in D55 at 0 and 12 h post-treatment, driving the synthesis of G-type and S-type lignins. Lignin derivatives contribute to cell wall reinforcement and oxidative stress mitigation. In the zma00073 pathway, *ZmCYP86A4* facilitated 16-hydroxypalmitate production and *ZmHHT1* catalyzed 16-feruloyloxypalmitate formation. ZmKCS mediated docosanedioate biosynthesis. *ZmCER1* (*Zm00001eb074170*) and *ZmWSD1* (*Zm00001eb308150*) showed constitutive activation in D55, enhancing cuticular permeability control. In the benzoxazinoid biosynthesis pathway, coordinated upregulation of *ZmBx2*, *ZmBx5*, *ZmBx8_9*, and *ZmBx6* in D55 at 12 h post-treatment promoted DIMBOA-glucoside production. The synergistic activation of these pathways in D55 establishes a multi-layered defense system, combining physical barriers (cuticular lipids), chemical deterrents (benzoxazinoids), and ROS-scavenging metabolites (phenylpropanoids). This integrated metabolic reprogramming underlies D55’s superior salt stress adaptation compared to D96.

### 2.9. Phytohormone Signaling Divergence Between D55 and D96 Under Salt Stress

Phytohormones orchestrate salt stress adaptation through growth regulation and stress signaling pathways [14]. Transcriptional profiling revealed genotype-specific hormonal strategies (Figure S4). At 4 h post-treatment, four genes involved in BR, GA, and ABA biosynthesis/signaling exhibited upregulation in D55 including *Zm00001eb155320* (IAA-amino acid hydrolase ILR1-like 4), *Zm00001eb110040* (Cytochrome P450 709B2), *Zm00001eb298190* (gibberellin 2-oxidase12), and *Zm00001eb300240* (nine-cis-epoxycarotenoid dioxygenase5). However, only one gene *Zm00001eb298620* (4-coumarate—CoA ligase-like 5) associated with jasmonic acid (JA) showed upregulated in D96. At 12 h post-treatment, seven hormone-related genes associated with JAS/GA/ABA/AUXIN were downregulated, and only one gene *ZmYUCCA4* (*Zm00001eb088950*) showed high expression levels in D55. At the late stage of salt stress (72 h), two genes, *Zm00001eb122610* (UDP-glycosyltransferase 73D1) and *Zm00001eb098150* (probable indole-3-pyruvate monooxygenase YUCCA3), sustained upregulation, which were associated with auxin biosynthetic enzymes. Limited response via *ZmYUCCA10* (*Zm00001eb096820*) activation was observed in D96. This temporal divergence highlights D55’s proactive hormonal reprogramming (early BR/GA/ABA activation, sustained auxin synthesis) versus D96’s reactive JA-dominated signaling. The coordinated multihormone strategy in D55 aligns with its enhanced plant growth and root architectural plasticity under salt stress (Figure 2).

### 2.10. Transcriptomic and Genetic Variation Analyses Identify Salt Stress-Responsive Candidate Genes

**Figure 6:**
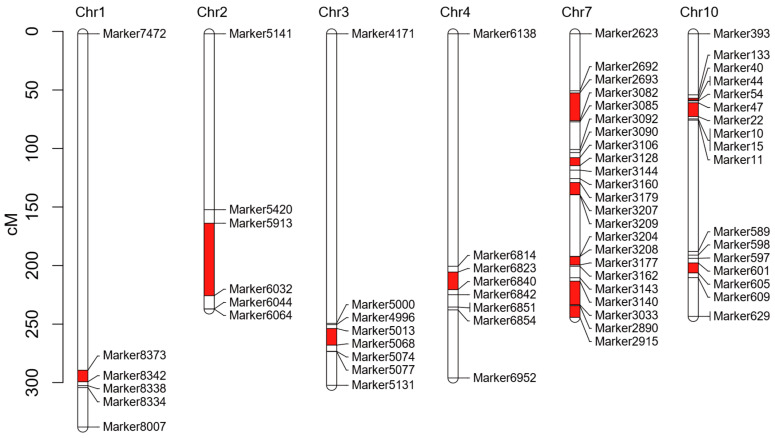
Distributions of differential chromosome segments between salt-tolerant genotype D55 and salt-sensitive genotype D96. The red frame shows the differential chromosome segments between D55 and D96.

To identify candidate genes associated with salt stress response, we previously conducted specific-locus amplified fragment sequencing on the BC_4_F_3_ population [32], revealing 11 divergent chromosomal segments between the salt-tolerant D55 and salt-sensitive D96 lines (Figure 6). These segments were distributed across chromosomes 1, 2, 3, 4, 7, and 10, with chromosome 7 exhibiting the highest number of divergent segments. Genes located within these segments were cross-referenced with DEGs identified from transcriptomic data under salt stress conditions. A total of 226 DEGs were identified within these segments; 106 were identified at 4 h, 138 at 12 h, and 132 at 72 h post-treatment, respectively. Fifty-two genes showed consistent differential expression across three time points (Table S8). Notably, nine transcription factors (TFs) demonstrated significant differential expression patterns, including *Zm00001eb098330* (WRKY74), *Zm00001eb099340* (ZIM 15), *Zm00001eb100710* (GeBP 8), *Zm00001eb100860* (bZIP 123), *Zm00001eb108730* (MYB 29), *Zm00001eb100380* (HEC2), *Zm00001eb100800* (ERF109), *Zm00001eb109040* (CCAAT-HAP2.4), and *Zm00001eb306690* (G2-like 15). These TFs, characterized by conserved domains, represent promising candidates for regulating salt stress responses. Their temporal expression patterns suggest potential roles in both early signaling and sustained stress adaptation mechanisms.

**Figure 7:**
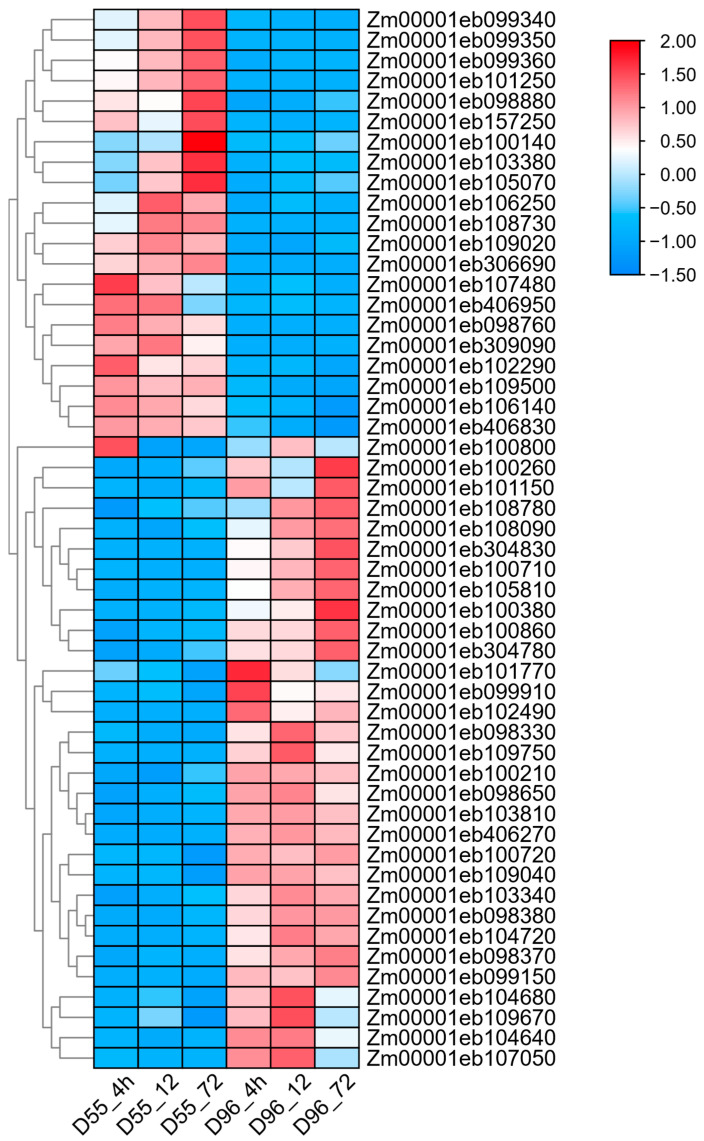
Heatmaps of 52 DEGs within differential chromosome segments in salt-tolerant D55 compared with salt-sensitive D96. A threshold of FDR < 0.05 and |log_2_FC| > 1 was used to screen DEGs between D55 and D96 after salt stress at 4, 12, and 72 h.

A clustering analysis and expression heatmap of the 52 consistent DEGs across three time points revealed distinct expression patterns (Figure 7). Twenty-one genes exhibited significantly higher expression in the salt-tolerant parent D55, suggesting potential roles as positive regulators of salt stress adaptation. Sixteen of these genes were localized to chromosome 2, with one gene on chromosome 3 and two genes each on chromosomes 7 and 10. Notably, three transcription factors including *Zm00001eb099340*, *Zm00001eb108730*, and *Zm00001eb306690* were identified within this subset.

SNP and InDel variations in the exon regions of these 21 candidate genes were analyzed using GATK [33], revealing nonsynonymous sequence variants in 14 candidate genes (Table S9). Thirteen genes harbored nonsynonymous single-nucleotide variants (SNVs), while *Zm00001eb406950* contained three nonframeshift substitutions and one nonsynonymous SNV. Notably, *Zm00001eb108730* exhibited eight nonsynonymous SNVs, and *Zm00001eb406830* contained four nonsynonymous SNVs, with other genes showing one to two nonsynonymous SNVs. Two transcription factors, *Zm00001eb099340* (ZIM 15) and *Zm00001eb108730* (MYB 29), were among the genes displaying these functional sequence variations, further supporting their potential involvement in salt stress response mechanisms.

## 3. Discussion

Soil salinization has emerged as a critical environmental constraint affecting global agricultural sustainability, with approximately 20% of irrigated croplands experiencing productivity declines due to salt accumulation [34]. Roots play a pivotal role in plant salt stress adaptation through sensing via membrane receptors, maintaining ionic/osmotic homeostasis, and remodeling adaptive growth. As a principal global food crop, maize (*Zea mays* L.) exhibits high sensitivity to salt stress, manifesting as suppressed seed germination, impaired seedling growth, and compromised reproductive development through ionic toxicity, osmotic imbalance, and oxidative damage [35]. While salt tolerance mechanisms in dent corn have been partially characterized, the molecular networks underlying salt stress responses in sweet corn remain underexplored, particularly in roots during seedling development. This study identified 14 candidate genes associated with salt stress adaptation in roots of sweet corn seedlings. These findings provide foundational insights into the molecular adaptations of sweet corn under saline conditions, offering new targets for improving salt tolerance through molecular breeding strategies.

### 3.1. Core Persistent DEGs in Salt Stress Response

Pairwise comparisons analysis identified 22 and 35 persistent DEGs in salt-tolerant D55 and salt-sensitive D96, respectively, with 8 shared genes likely mediating fundamental salt adaptation mechanisms (Figure 3). Functional annotation revealed two chromatin regulators, *Zm00001eb148070* (regulator of chromosome condensation 1) and *Zm00001eb367810* (RCC1 family protein), potentially influencing hypoxia-responsive chromatin remodeling. This aligns with dynamic chromatin accessibility adjustments at hypoxia-responsive gene promoters in *Arabidopsis* during early hypoxia stress, and flexible regulation of chromatin aggregation is the key to plant adaptation to transient hypoxia [36]. *Zm00001eb228670* encodes MYB-related transcription factor 96, and *ThMYB8* can reduce oxidative damage via ROS metabolic regulation to enhance salt stress tolerance [37]. *Zm00001eb268600* (extended protein analogue) is implicated in cell wall remodeling, and its persistent upregulation may alleviate mechanical stress by enhancing cell wall ductility. *Zm00001eb372500* (HVA22-like protein), as a component of the ABA signaling pathway, may contribute to stomatal regulation and energy metabolism reprogramming [38]. These core DEGs may be indispensable in the modulation of salt stress in sweet corn.

A total of 49 persistent DEGs, comprising two genotype-specific DEGs and eight common DEGs of D55 and D96, were significantly enriched in pathways associated with cellular amino acid metabolism, carboxylic acid metabolism, and organic acid metabolism (Table S6). These metabolic processes may support energy supply under hypoxic conditions through the recirculation of tricarboxylic acid cycles, while also regulating the synthesis of osmoregulatory substances. Notably, the sustained activation of small-molecule metabolic processes may play a role in the biosynthesis of antioxidant compounds, which are crucial for mitigating ROS bursts induced by salt stress [39,40]. The coordinated regulation of these metabolic pathways likely represents a fundamental adaptive mechanism for maize in response to salt stress.

### 3.2. ROS Homeostasis Enhancement Underpins Salt Tolerance in D55

Salt tolerance is fundamentally linked to the spatiotemporal regulation of ROS generation and scavenging systems [41]. Our analysis revealed that the salt-tolerant line D55 initiated rapid upregulation of *Zm00001eb070310* (polyamine oxidase, PAO) within 4 h post-treatment. This enzyme catalyzes polyamine degradation to generate ROS, potentially serving as an early redox signaling trigger [42]. In contrast, the salt-sensitive line D96 exhibited delayed induction of *Zm00001eb341910* (respiratory burst oxidase homolog, RBOH) at 12 and 72 h, consistent with the NADPH oxidase-mediated sustained ROS production mechanism under salt stress [43]. This temporal divergence suggests that D55 prioritizes early ROS-mediated signaling to coordinate stress response cascades.

The robust antioxidant capacity of D55 was further evidenced by three *superoxide dismutase* (SOD) genes (*Zm00001eb378880*, *Zm00001eb226600*, and *Zm00001eb420210*) maintaining sustained upregulation from 4 to 72 h, aligning with their established role as primary scavengers of superoxide radicals [44]. Notably, these *SOD* genes showed no significant induction in D96, highlighting genotype-specific regulatory divergence. Complementary phased activation of glutathione-S-transferase (*GST*, *Zm00001eb021620* and *Zm00001eb282430*) and peroxidase (*POD*, *Zm00001eb047120*) in D55 at 12/72 h supports their involvement in secondary detoxification via glutathione recycling and phenolic oxidation, consistent with earlier reports in *Brassica juncea* L. [45]. Trypan blue staining confirmed significantly reduced cell death in D55, directly correlating with its enhanced ROS scavenging efficiency, which is consistent with a phenomenon paralleling AMF-mediated salt tolerance mechanisms in cotton [46].

This study identified constitutive high expression of *Zm00001eb074210* (plasma membrane intrinsic protein 1) in D55. It has been reported that PIP1 and PIP2 regulate water absorption and transport, maintain cell turgor pressure and organ morphology, and mediate H_2_O_2_ transmembrane diffusion. PIP is necessary for extracellular H_2_O_2_ transport to the cytoplasm [47]. PIP also participates in root morphogenesis and root morphogenesis, essential for taproot elongation and lateral root development [48]. The high expression of *ZmPIP1a* in D55 likely enables the rapid establishment of redox equilibrium during acute salt stress by facilitating H_2_O_2_ compartmentalization, providing novel insights into spatiotemporal regulation of ROS signaling.

While our findings illuminate ZmPIP1a’s potential role in salt adaptation, the molecular mechanisms coordinating PIP-mediated ROS homeostasis with other stress-responsive pathways remain unresolved. Future studies should investigate whether *ZmPIP1a* participates in H_2_O_2_-triggered calcium signaling or modulates ion transporter activity to counteract Na^+^ toxicity. However, how these key genes regulate abiotic stress to maintain efficient ROS homeostasis remains to be further explored.

### 3.3. Lignin, Cutin/Suberin/Wax, and Benzoxazine Biosynthesis Promote Salt Tolerance

Lignin, cutin/suberin/wax, and benzoxazine biosynthetic pathways play key roles in the complex regulatory network of plant response to salt stress [49,50,51]. Transcriptomic profiling revealed significant enrichment of DEGs within three critical pathways: phenylpropanoid metabolism (zma00940), cutin/suberin/wax biosynthesis (zma00073), and benzoxazinoid biosynthesis (zma00402). D55 exhibited upregulated expression of *Zm00001eb041100*, *Zm00001eb249960,* and *Zm00001eb146710*, which drive 16-hydroxypalmitate, 16-feruloyloxypalmitate, and docosanedioate biosynthesis—core precursors for cutin monomers [52]. At 12 h post-salt treatment, coordinated induction of *Zm00001eb074170*, *Zm00001eb308150,* and *Zm00001eb039570*, enhanced cutin/suberin transmembrane transport and oxidative polymerization, facilitating apoplastic barrier formation in roots [53].

The benzoxazinoid biosynthesis pathway was uniquely activated in D55, with *Zm00001eb165580* (Bx5), *Zm00001eb304070* (UGT76C1), *Zm00001eb164860* (BX6), and *Zm00001eb165620* (Bx2) synergistically upregulated to promote DIMBOA-glucoside biosynthesis. This endogenous phytoanticinin mitigates salt-induced damage through ROS chelation and suppression of plasma membrane ion leakage [51,54]. Sweet corn orchestrates a hierarchical salt defense system by synchronizing lignin deposition, cuticular barrier reinforcement, and benzoxazinoid-mediated ROS detoxification. This metabolic coordination provides novel insights for improving salt tolerance via pyramiding these complementary mechanisms in maize breeding programs.

### 3.4. Temporal Dynamics of Phytohormone Signaling in Salt Stress Adaptation

**Figure 8:**
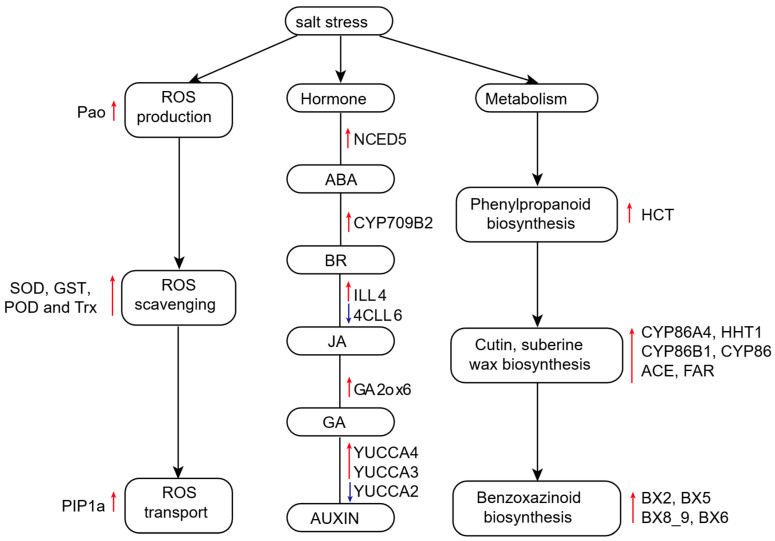
A hypothetical model of the response mechanism for salt stress in sweet corn seedlings. The red arrows indicate upregulated gene expression in the tolerant sweet corn genotype D55, and the blue arrows indicate the upregulated gene expression in the sensitive sweet corn genotype D96.

Phytohormones orchestrate salt stress responses through interconnected signaling networks that regulate physiological adjustments and transcriptional reprogramming [16,55]. Our comparative analysis revealed distinct hormone response patterns between D55 and D96. At 4 h post-salt treatment, D55 exhibited coordinated upregulation of BR, GA, ABA, and AUXIN biosynthesis/signaling genes (Figure 8), potentially enhancing cell wall remodeling and ion homeostasis through BR-GA crosstalk. The synergistic activation of BR and ABA signaling likely improved stomatal regulation and osmotic adjustment [56], while sustained AUXIN signaling maintained root meristem activity to facilitate stress adaptation [57]. The OsCSLD4 gene enhances salt tolerance by regulating ABA synthesis in rice [58]. In addition, the jasmonic acid signaling pathway and melatonin are also involved in the response of rice to salt stress [58,59]. These responses correlated with D55’s superior phenotypic performance in seedling height, root architecture, biomass, and chlorophyll retention under salinity (Figure 1B).

The sustained induction of YUCCA4 (*Zm00001eb088950*) in D55 at 12 h post-treatment indicated persistent auxin biosynthesis via indole-3-pyruvate conversion [60]. This AUXIN-ABA-GA regulatory nexus may optimize resource allocation by balancing root growth promotion with shoot growth restriction [61]. Notably, the suppression of seven JA/BR/GA/ABA-related genes in D55 suggests temporal antagonism between hormone pathways, potentially prioritizing energy-efficient stress responses [62]. At 72 h, D55 maintained hormone homeostasis through UDP-glycosyltransferase 73D1-mediated hormone inactivation and YUCCA3 (*Zm00001eb098150*)-driven auxin replenishment [63]. These compensatory mechanisms highlight the critical balance between hormone synthesis, modification, and degradation during prolonged stress (Figure 8). The root system plays a core role in the response to salt stress by adjusting gene expression, structural development, and signal networks [8,14]. In this study, we established a model of a root system responding to salt stress in sweet corn (Figure 8).

In this study, we identified fourteen candidate genes orchestrating complementary salt tolerance mechanisms. Four genes are associated with ROS scavenging. *Zm00001eb100140* encodes photosystem I chlorophyll a/b-binding protein 6, chloroplastic, which stabilizes photosynthetic electron transport to mitigate photoinhibition [64]. *Zm00001eb102290* regulates mitochondrial NADPH recycling and plays a key role in redox homeostasis [65]. *Zm00001eb103380*, as a key enzyme in detoxification metabolism, may alleviate salt-induced lipid peroxidation damage by hydroxylating secondary metabolites [66]. *Zm00001eb406830* encodes endonuclease III homolog 1, chloroplastic, which may be involved in repairing oxidative chloroplast DNA damage [67]. *Zm00001eb109500* (CDPK-related kinase 5) and *Zm00001eb406950* (B3 domain-containing protein) may be involved in osmotic regulation by cascade phosphorylating downstream target proteins through calcium signaling [68]. *Zm00001eb101250* (isoamylase3-like1) may alleviate Fe^3+^ chelation imbalance caused by salt stress [69]. This hierarchical network integrates photoprotection, ROS scavenging, osmoregulation, ion homeostasis, and DNA repair mechanisms (Figure 8). The functional convergence of these pathways underscores the system-level complexity of salt adaptation, providing prioritized targets for molecular breeding programs.

## 4. Materials and Methods

### 4.1. Plant Materials and Salt Treatment

A sweet corn BC_4_F_3_ population [32] was utilized for salt tolerance screening. Three uniform seedlings at the two-leaf and one-heart stages from each line were subjected to 150 mM NaCl treatment for 72 h. Phenotypic parameters, including shoot height and root length, were measured to identify extreme responders. Two contrasting CSSLs were selected based on divergent phenotypic responses to salinity: salt-tolerant D55 and salt-sensitive D96 (Table S10). The seeds of D55 and D96 were surface-sterilized with 0.1% sodium hypochlorite (15 min), rinsed thrice with distilled water, and germinated in nursery substrate. Seedlings at the two-leaf and one-heart stages were randomly allocated into two groups: control (CK, Hoagland solution) and salt-treated (150 mM NaCl-supplemented Hoagland solution). Two groups of plants were cultivated in six planting boxes, with three designated as control (CK) and the other three treated with 150 mM NaCl. These boxes were maintained in a growth chamber under controlled conditions (26 °C, 70% relative humidity, 14 h light/10 h dark cycle). Whole root tissues from three plants per replicate were sampled at 0, 4, 12, and 72 h post-treatment for transcriptome analysis, with three biological replicates per line collected at each time point. Samples were immediately frozen in liquid nitrogen and stored at −80 °C.

### 4.2. Phenotype Observation After Salt Stress

A randomized complete block design with three biological replicates was implemented. Samples from control and salt-treated groups (72 h) were collected for phenotypic evaluation. Ten plants per replicate were analyzed for fresh weight, shoot height, root length, and leaf SPAD values using a handheld SPAD-502 m (Konica Minolta, Tokyo, Japan). Root architecture was quantified via high-resolution scanning (Epson Perfection V3.771, Epson, Nagano, Japan); this was followed by WinRHIZO v2.0 software analysis (Regent Instruments Inc., Quebec City, QC, Canada) to determine total root area and morphological parameters. For cell viability assessment, salt-stressed leaves were immersed in 0.4% (*w*/*v*) trypan blue solution (Plant Tissue Stain Kit, Solarbio Co., Ltd., Beijing, China) for 1 h at 25 °C. Subsequently, tissues were destained in 1.25 g/mL chloral hydrate solution for 12 h with daily solution replacement to remove nonspecific staining. The roots of untreated plants were utilized as controls. Cell death was assessed using a stereo microscope.

### 4.3. Antioxidant Activity Determination After Salt Stress

Leaf and root tissues from each sample were utilized for antioxidant enzyme analysis and malondialdehyde (MDA) quantification. Fresh tissues (0.2 g) were homogenized in ice-cold 62.5 mM phosphate buffer (pH 7.8, 1.0% *w*/*v* polyvinylpyrrolidone) using a pre-chilled mortar. Homogenates were centrifuged at 12,000× *g* (4 °C, 10 min), with supernatants collected for biochemical assays. Catalase (CAT), peroxidase (POD), and superoxide dismutase (SOD) activities were quantified using commercial ELISA kits (ZK-L0424, ZK-L0423, ZK-L0426; Ziker Biotechnology Co., Ltd., Shenzhen, China) following manufacturer protocols. MDA content was determined via an enzyme-linked immunoassay assay using the Plant MDA ELISA Kit (ZK-P7111, Ziker Biotechnology, Shenzhen, China), with absorbance measured at 450 nm.

### 4.4. RNA Extraction, Library Preparation, and Sequencing

RNA sequencing was conducted by Personal Biotechnology Co., Ltd. (Shanghai, China). Total RNA was isolated using the HiPure Plant RNA Maxi Kit (Magen Biotech, Shanghai, China) following manufacturer protocols. RNA integrity was verified through 0.8% agarose gel electrophoresis and spectrophotometric analysis (NanoDrop 2000, Thermo Scientific), with high-quality samples (28S:18S ratio > 1.5, A260/A280 = 1.8–2.2) selected for library construction. Ribosomal RNA was filtered using the Illumina Ribo Zero rRNA Removal Kit (San Diego, CA, USA). mRNA was enriched from 3 μg total RNA via poly-T oligo-attached magnetic beads. A total of 24 sequencing libraries were prepared using the TruSeq Stranded mRNA Library Prep Kit (Illumina, San Diego, CA, USA), with cDNA fragment sizes (~200 bp) validated by an Agilent 2100 Bioanalyzer (High Sensitivity DNA Kit, Palo Alto, CA, USA). Paired-end sequencing (2 × 150 bp) was performed on an Illumina NextSeq500 platform. Raw reads were processed to remove (1) adapter-contaminated sequences, (2) low-quality reads (Q20 < 90%), and (3) short reads (<50 bp). All raw data have been deposited in the Genome Sequence Archive (GSA) under accession number CRA040949 at the National Genomics Data Center (NGDC).

### 4.5. RNA Sequencing Data Analysis

Raw reads underwent quality filtering (Q > 20) prior to alignment against the reference genome (Zea_mays.B73_RefGen_v5.dna.toplevel.fa) from the Plants Ensembl database (https://ftp.ensemblgenomes.ebi.ac.uk/pub/plants/release-58/fasta/zea_mays/, accessed on 16 October 2023). The reference genome index was created using Bowtie2 v2.4.2 [70] with the default parameters. Read mapping and splice junction detection were executed via TopHat2 v2.1.1 [71], allowing ≤ 2 mismatches per read. Gene annotation was performed by integrating data from multiple databases: NCBI Nucleotide (NT), Gene Ontology (GO), Enzyme Commission (EC), Kyoto Encyclopedia of Genes and Genomes (KEGG), and Swiss-Prot. The read count for each gene was determined using HTSeq as the original gene expression level [72]. Expression levels were normalized as reads per kilobase per million mapped reads (RPKM), with RPKM > 1 considered expressed [73]. Differential expression analysis employed DESeq v1.48.0 [74], and genes meeting |log_2_FC| > 1 and false discovery rate (FDR) < 0.05 were classified as DEGs. Functional enrichment analysis of DEGs was conducted using topGO v2.42.0 [75] for GO terms and KAAS v2.1 [76] for KEGG pathway terms.

### 4.6. Quantitative Real-Time PCR

A total of 6 DEGs were chosen randomly for qRT-PCR analyses to validate the sequencing data. Gene-specific primer pairs (Table S11) were designed using Primer Premier 5.0 software [77]. Total RNA was isolated from frozen root tissues using the RNAprep Pure Plant Kit (Tiangen Biotech Co., Ltd., Beijing, China) following manufacturer protocols. RNA purity and integrity were verified via NanoDrop 2000 spectrophotometry (Thermo Scientific, Waltham, MA, USA) with RNase-free water as the blank control. cDNA synthesis was performed using FastKing gDNA Dispelling RT SuperMix (Tiangen Biotech Co., Ltd., Beijing, China), with the housekeeping gene GAPDH [78] serving as the endogenous control. Relative gene expression was calculated using the 2^−ΔΔCT^ method [79].

### 4.7. Combined Analysis of Differential Chromosomal Fragments and Transcriptome

The D55 and D96 genotypes were identified from the BC_4_F_3_ population, which was utilized to develop a high-density genetic linkage map. A consensus map spanning 2413.25 cM was constructed using 3876 single-nucleotide polymorphism (SNP) markers distributed across 10 chromosomes, with an average marker interval of 0.62 cM [32]. Polymorphic markers distinguishing D55 and D96 were analyzed using Mapchart 2.2 [80] to visualize divergent chromosomal segments. These genes located within the differential chromosomal fragments were identified using the maizeGDB database. By integrating the DEGs obtained from transcriptome data, candidate genes exhibiting significant changes at 4, 12, and 72 h post-salt stress (FDR < 0.05, |log_2_FC| > 1) were filtered from the differential chromosome segments. This integrative strategy facilitated the identification of candidate genes potentially involved in salt stress adaptation.

### 4.8. Variation Analysis of Candidate Genes

The detection and filtration of SNPs and insertion–deletion (InDel) markers were performed using the SelectVariants and VariantFiltration modules in GATK [33]. For SNP identification, the following filtering criteria were applied: Q score > 20, read depth > 8, variant-supporting reads > 2, and *p*-value < 0.01. Identical quality thresholds were implemented for InDel markers, including Q score > 20, read depth > 8, variant-supporting reads > 2, and *p*-value < 0.01. High-confidence SNP and InDel variants meeting these stringent criteria were subsequently selected for downstream analyses.

### 4.9. Statistics

Statistical analyses were performed using SPSS version 17.0 with significant differences determined by Student’s *t*-test or two-way ANOVA followed by Dunnett’s multiple comparisons test. Gene expression patterns were visualized through heatmaps generated by TBtools V2.225 [81].

## 5. Conclusions

Our study demonstrates distinct phenotypic, physiological, and transcriptomic responses to salt stress between two sweet corn CSSLs. The salt-tolerant line D55 exhibited superior performance in terms of seedling height, root length, seedling weight, relative chlorophyll content, and ROS-scavenging enzyme activities, accompanied by high expression levels of genes associated with ROS scavenging, phenylpropanoid metabolism, and benzoxazinoid biosynthesis. Integrative analysis of transcriptomic and SLAF-seq data identified 14 high-confidence candidate genes. These findings elucidate a coordinated regulatory model in sweet corn salt adaptation, where hormone signaling cascades, ROS homeostasis, and specialized metabolite biosynthesis synergistically enhance stress resilience. Specifically, auxin-mediated root plasticity, phenylpropanoid-driven apoplastic barrier formation, and benzoxazinoid-mediated redox buffering collectively mitigate ionic and osmotic stress damage. Our work provides novel insights into the molecular mechanisms underlying salt tolerance in sweet corn and delivers key candidate genes for marker-assisted breeding.

## Figures and Tables

**Table 1:** Genes involved in the reactive oxygen species homeostasis between D96 and D55 under salt treatment.

Type	Enzyme	Gene Name	log_2_FC (D96 vs. D55)		
			4 h	12 h	72 h
ROS production	PAO	*Zm00001eb070310*	1.25		
	RBOH	*Zm00001eb341910*	−5.56	−5.53	−6.67
		*Zm00001eb410380*	−1.86		
ROS scavenging	SOD	*Zm00001eb378880*	2.06	1.88	3.06
		*Zm00001eb226600*		1.78	1.59
		*Zm00001eb420210*	1.61	2.68	2.82
	GST	*Zm00001eb141080*			1.37
		*Zm00001eb002780*			−1.11
		*Zm00001eb402630*			−1.22
		*Zm00001eb404560*	−1.97	−2.98	−1.87
		*Zm00001eb021720*		−1.37	
		*Zm00001eb021620*			−1.05
	POD	*Zm00001eb282430*			1.49
		*Zm00001eb222560*	5.74	6.47	9.59
		*Zm00001eb330530*			−1.46
		*Zm00001eb225230*	−1.15		−1.04
		*Zm00001eb047120*		1.23	
	Trx	*Zm00001eb201870*			−2.90
ROS transport	PIP	*Zm00001eb074210*	3.49	4.40	7.10

ROS: reactive oxygen species. A threshold of FDR < 0.05 and |log_2_FC| > 1 was used to screen DEGs between D96 and D55 after salt stress at different time points. Blanks indicate nonsignificant difference. Red and blue indicate a relative increase and decrease in expression (log_2_FC) in comparisons between D96 and D55 after salt treatment, respectively.

## Data Availability

Data are contained within this article or Supplementary Materials.

## Supplementary Materials

The following supporting information can be downloaded at https://www.mdpi.com/article/10.3390/plants14111687/s1, Figure S1. Principal component analysis (PCA) plot revealed the variance among each group; Figure S2. Validation of expression patterns of DEGs at 4 h and 12 h by RT-qPCR assay. (A) qRT-PCR verification of 6 differentially expressed genes (DEGs) in sweet corn seedlings at 4 h and 12 h after salt treatment, respectively. The total RNAs used for the transcriptome profile analyses by RNA-Seq were used in the RT-qPCR assay, including three biological replicates. The relative expression levels of the selected genes were calculated using the 2^−ΔΔCT^ method. The *y*-axis represents the fold change in comparisons between D96 and D55 after salt treatment, respectively. The positive direction of the *y*-axis indicates upregulation and the negative direction of the *y*-axis indicates downregulation in the tolerant genotype D55. Error bars represent the standard deviation of the mean fold change values. (B) The expression correlation between the qRT-PCR and RNA-seq of the 12 DEGs between D96 and D55; Figure S3. GO and KEGG enrichment analysis of DEGs in salt-tolerant genotype D55 compared with salt-sensitive D96. (A) GO enrichment analysis of the DEGs between D55 and D96 at 0 h of salt stress; (B) GO enrichment analysis of the DEGs between D55 and D96 at 4 h of salt stress; (C) GO enrichment analysis of the DEGs between D55 and D96 at 12 h of salt stress; (D) GO enrichment analysis of the DEGs between D55 and D96 at 72 h of salt stress; (E) KEGG enrichment analysis of the DEGs between D55 and D96 at 0 h of salt stress; (F) KEGG enrichment analysis of the DEGs between D55 and D96 at 72 h of salt stress. Rich factor is the ratio of the DEG numbers in the corresponding pathway to the total annotated gene numbers in this pathway; Figure S4. Heatmaps of DEGs involved in hormone synthesis and signaling pathway in salt-tolerant D55 compared with salt-sensitive D96 under salt stress. A threshold of FDR < 0.05 and |log_2_FC| > 1 was used to screen DEGs between D55 and D96 at different times after salt stress. Red and blue indicate relatively high and low in expression level (FPKM, data scaled) in comparisons between D55 and D96 at 0 h, 4 h, 8 h, and 72 h after treatment, respectively. JA, Jasmonate. BR, Brassinosteroids. GA, Gibberellin. ABA, abscisic acid; Table S1: The comparison of the SOD, CAT, and POD activities and malondialdehyde (MDA) contents of the leaves and roots of D55 and D96 at different times after salt treatment; Table S2: The quality of the assembled transcripts; Table S3: The FPKM values of all expressed genes in each sample; Table S4: Lists of differentially expressed genes (DEGs) in a pairwise comparison of 4 samples in D55 (D55_0h, D55_4h, D55_12h, and D55_72h) and D96 (D96_0h, D96_4h, D96_12h, and D96_72h), respectively; Table S5: The common differentially expressed genes (DEGs) in D55 and D96; Table S6: The top 20 GO terms enriched by 49 common DEGs in D55 and D96; Table S7: Lists of differentially expressed genes (DEGs) identified at four distinct time points between D96 and D55; Table S8: Lists of DEGs within 11 divergent chromosomal segments between D96 and D55 at 4 h, 12 h, and 72 h post-treatment; Table S9: Analysis of genetic variation in 14 candidate genes for salt tolerance; Table S10: The seedling height and root length of a sweet corn BC4F3 population under salt stress; Table S11: Primers used for qRT-PCR analysis.
