# Supplementary figures and images for: Root Transcriptome Analysis Identifies Salt-Tolerance Genes in Sweet Corn Chromosome Segment Substitution Lines (CSSLs)
Source: Plants (Basel). 2025 May 31;14(11):1687. doi: 10.3390/plants14111687 (PMC12157856; doi:10.3390/plants14111687)

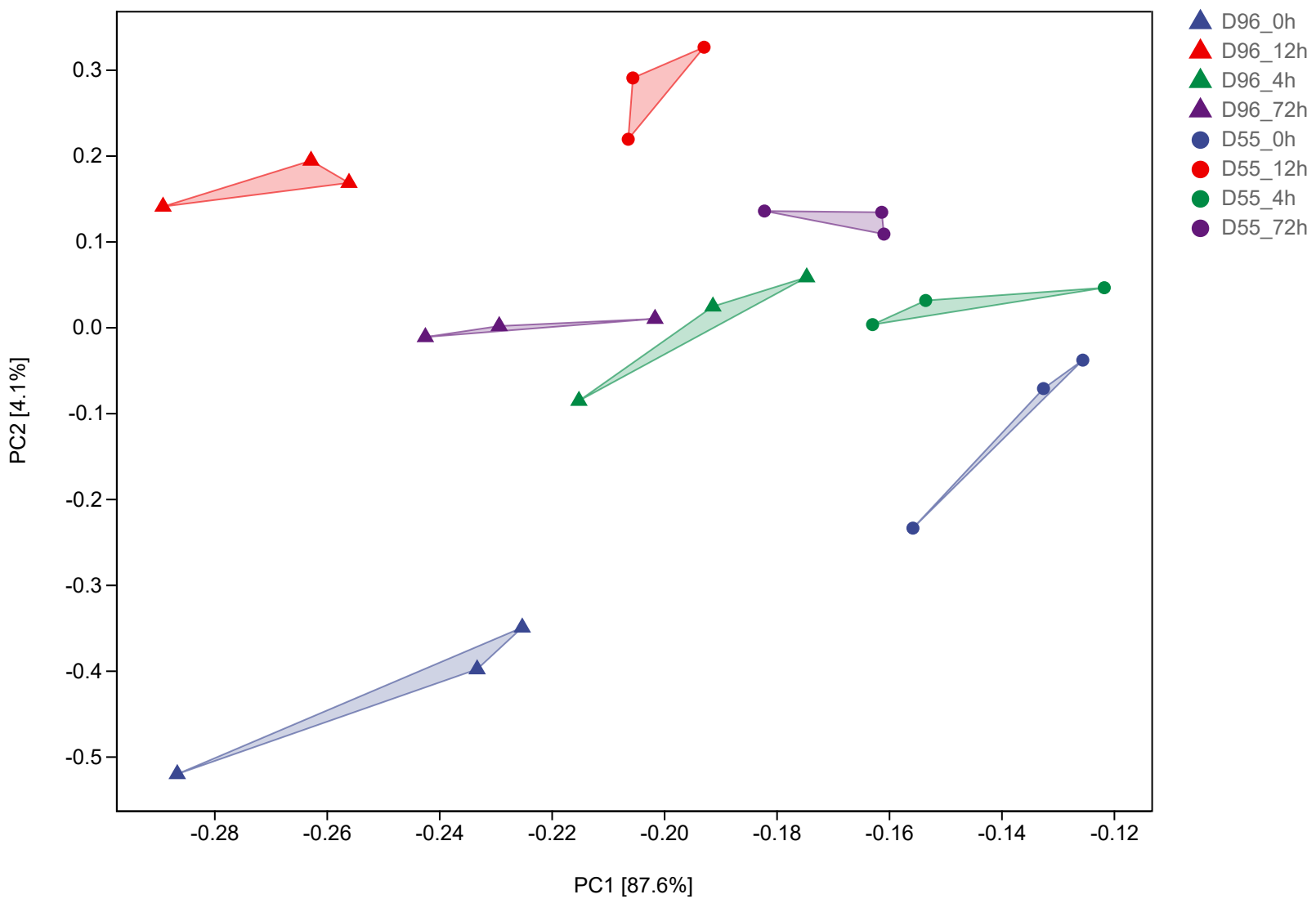

Supplement: Supplementary file 1 [file plants-14-01687-s001.zip › Figure S1.pdf]

A

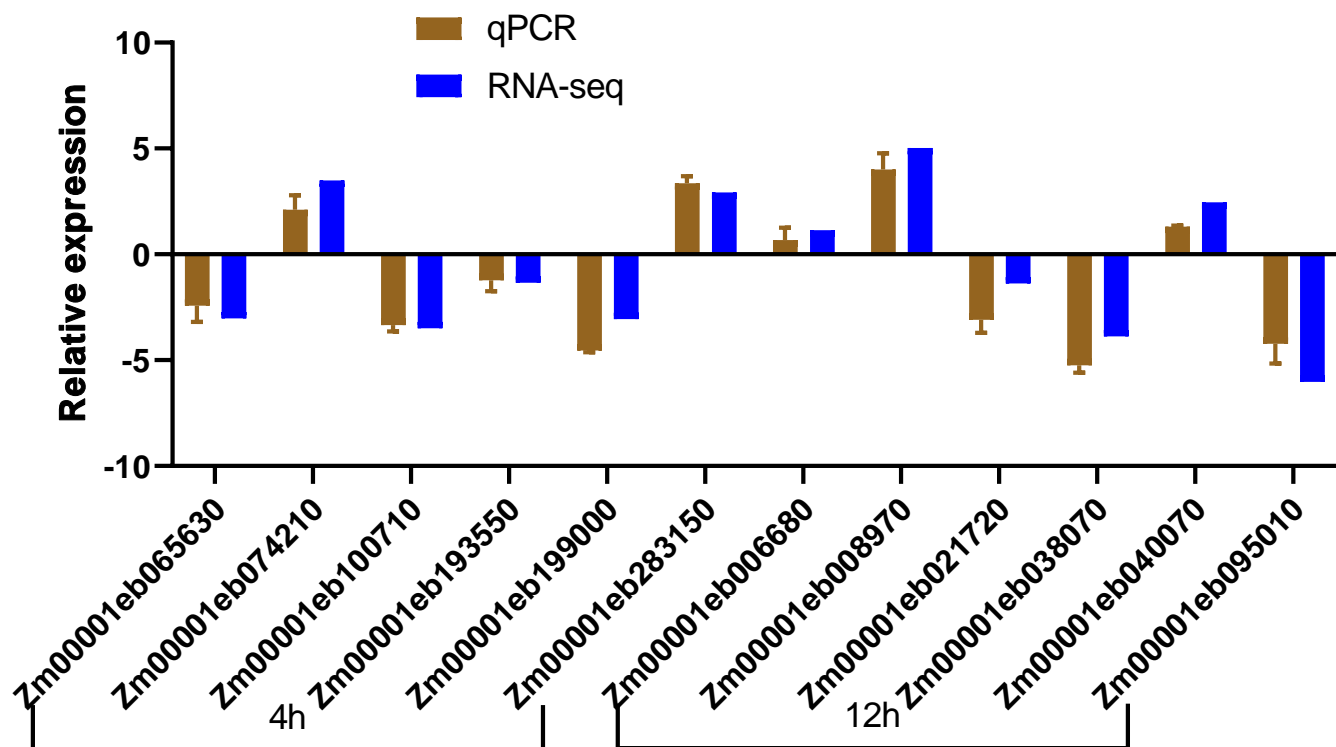

B

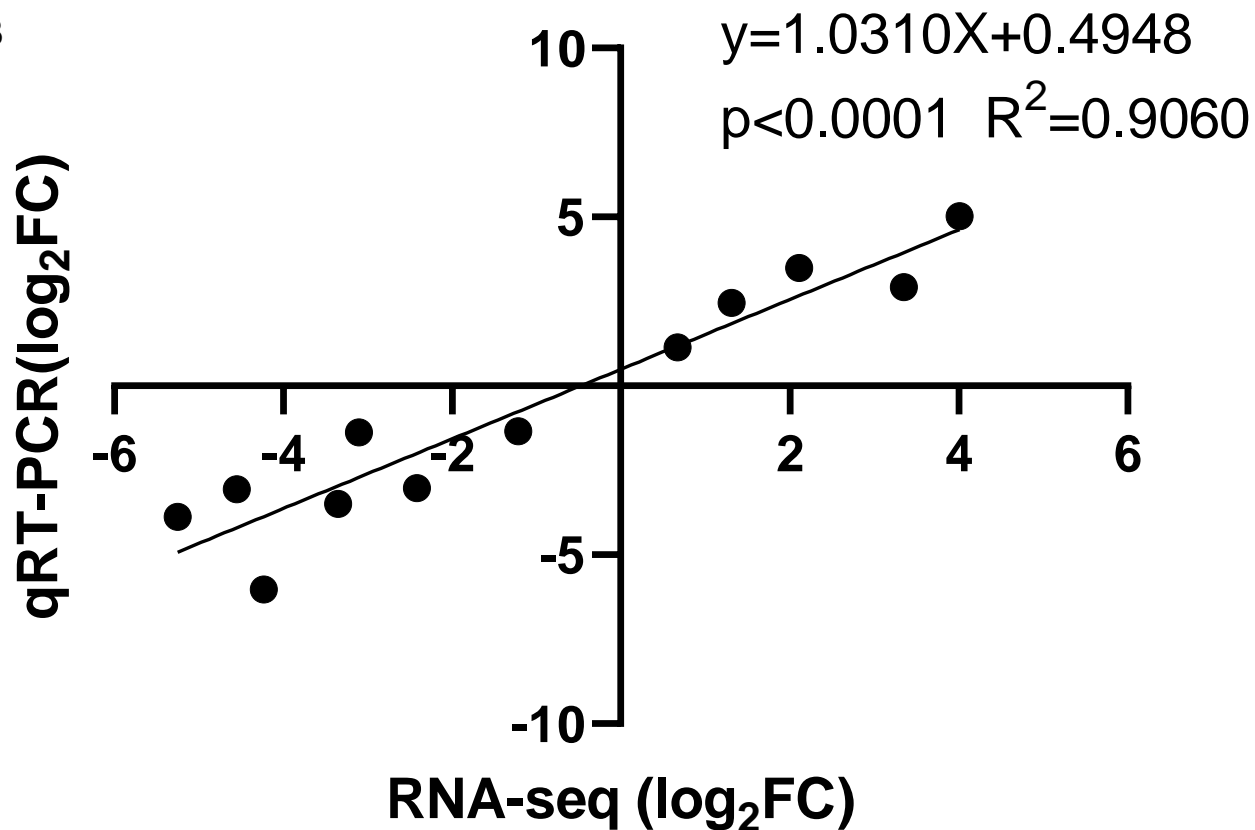

Supplement: Supplementary file 1 [file plants-14-01687-s001.zip › Figure S2.pdf]

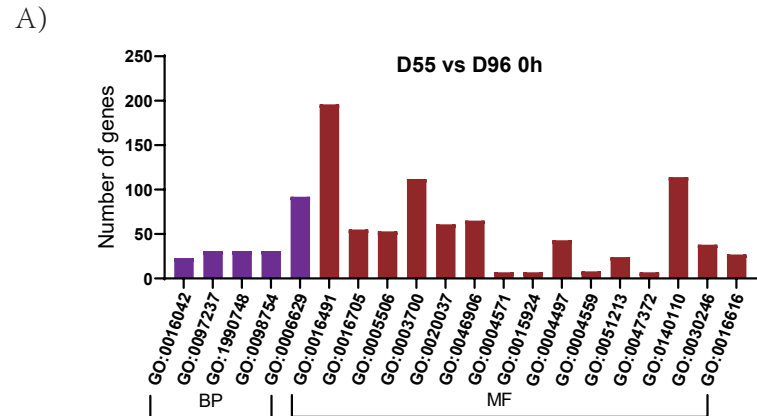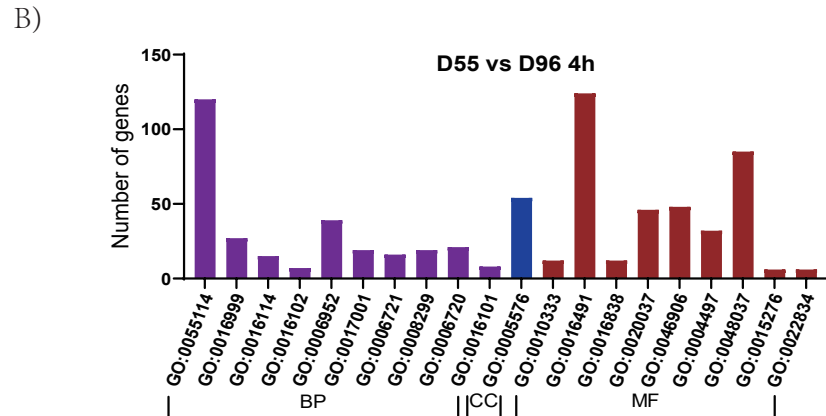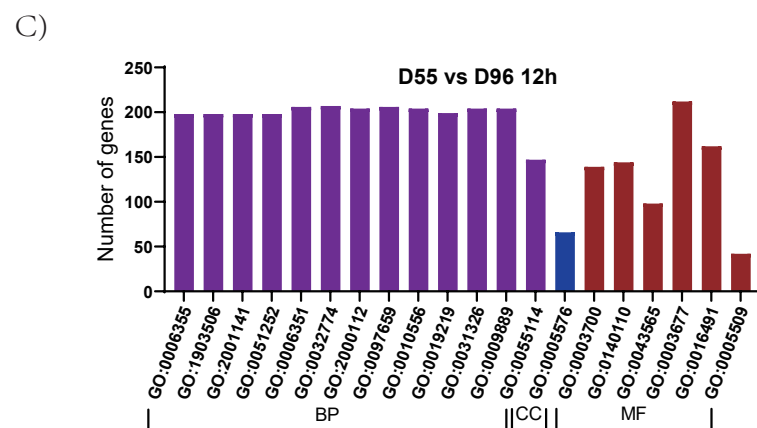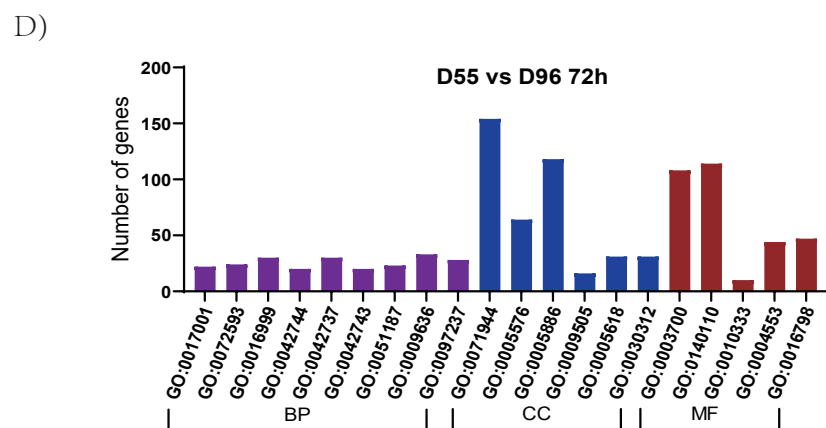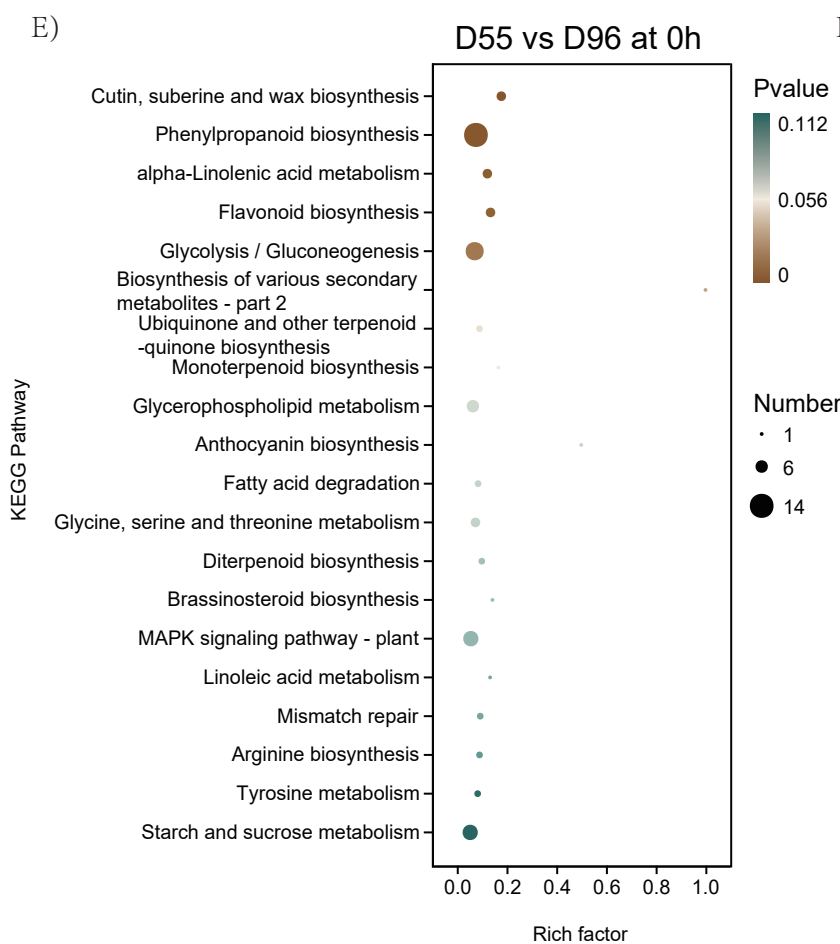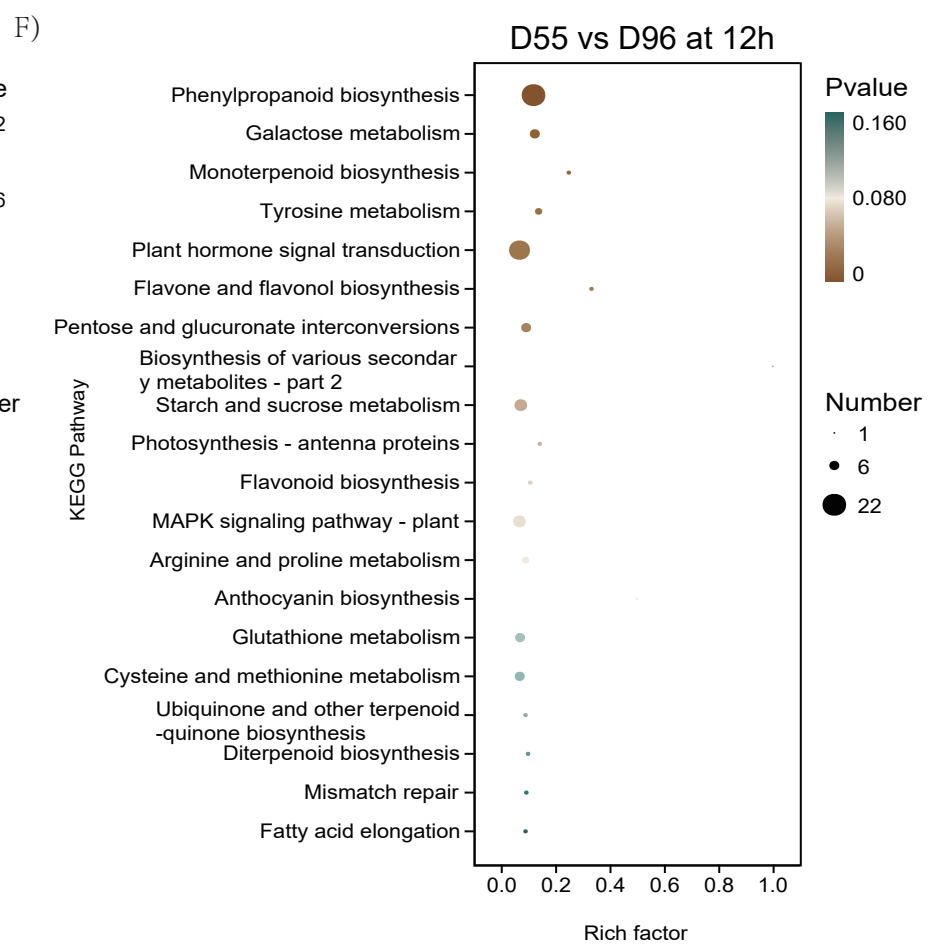

Supplement: Supplementary file 1 [file plants-14-01687-s001.zip › Figure S3.pdf]

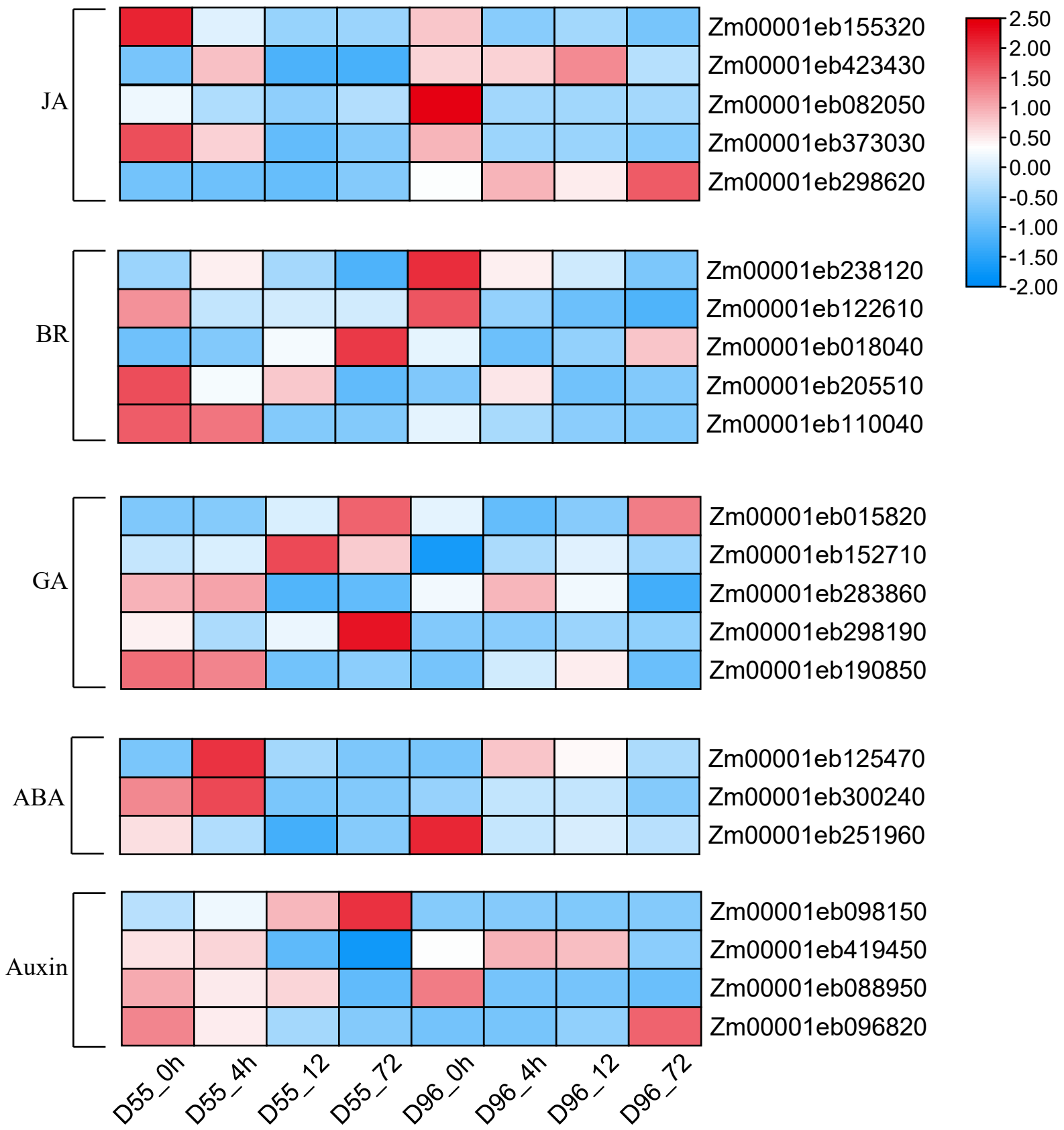

Supplement: Supplementary file 1 [file plants-14-01687-s001.zip › Figure S4.pdf]
